# Sacubitril/valsartan in a wide spectrum of heart failure patients (from mechanisms of action to outcomes in specific populations)

**DOI:** 10.1007/s10741-024-10471-1

**Published:** 2025-01-07

**Authors:** Przemyslaw Rajzer, Jan Biegus

**Affiliations:** 1 University of Glasgow School of Medicine, Glasgow, Scotland UK; 2 Institute of Heart Diseases, Wroclaw Medical University, Wroclaw, Poland

**Keywords:** ARNI, Sac/Val, Heart failure, HFrEF, HFpEF

## Abstract

Heart failure (HF) represents a significant global health challenge, characterized by high morbidity and mortality rates, decreased quality of life and a significant financial and economic burden. The prevalence of HF continues to rise, driven by an ageing population and an increasing burden of comorbidities such as hypertension, diabetes and obesity. Understanding the complex pathophysiology and developing effective treatments are critical for improving patient outcomes, yet the range of effective, life-prolonging medication classes has remained mostly constant in the last few decades. The introduction of angiotensin receptor neprilysin inhibitors (ARNI) was a major breakthrough in HF management, for the first time targeting the natriuretic peptide system in addition to the renin–angiotensin–aldosterone pathway to potentiate the effects of older drug classes. ARNI shows superiority in clinical outcomes compared to previous guideline-directed therapies, especially in patients with reduced ejection fraction (EF). It has now been implemented into international guidelines, endorsing its use in patients with HF and reduced ejection fraction (HFrEF) and HF with mildly reduced ejection fraction (HFmrEF). This review summarises the mechanism of action of Sac/Val, presents key clinical trials in a range of patient populations and HF aetiologies and outlines gaps in knowledge and potential novel uses of Sac/Val.

## Introduction

Heart failure (HF) encompasses a clinical syndrome characterized by impaired cardiac structure and function, with varied underlying causes, each associated with specific risk factors and requiring targeted management [1, 2]. It is broadly classified into heart failure with reduced ejection fraction (HFrEF), heart failure with mildly reduced ejection fraction (HFmrEF) and heart failure with preserved ejection fraction (HFpEF), each presenting unique features but sharing common pathophysiological mechanisms. Whilst HFrEF is characterised by sympathetic overdrive along with overactivation of the renin–angiotensin–aldosterone system (RAAS) and natriuretic peptide system (NPS), metabolic inflammation, endothelial dysfunction and cardiomyocyte hypertrophy and fibrosis are more substantial features in heart failure with preserved ejection fraction (HFpEF) [3]. Additionally, both HFrEF and HFpEF may present with myocardial remodelling processes, though the extent and nature of such changes can differ. These pathophysiological changes can manifest as congestion and features like dyspnoea, orthopnoea, peripheral oedema and reduced exercise tolerance [4].

**Fig. 1:**
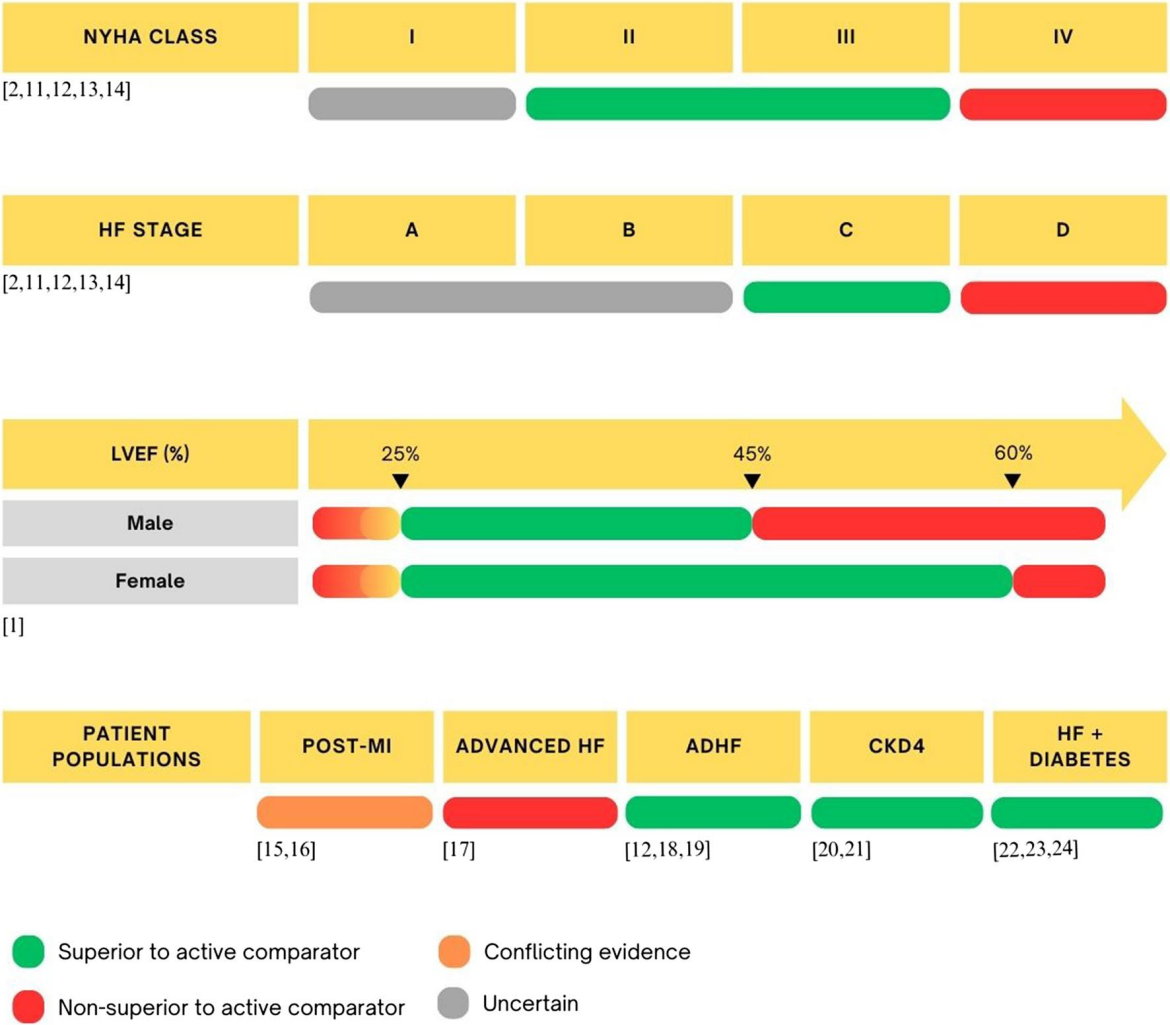
Summary of Sacubitril/Valsartan’s efficacy in various heart failure clinical patient populations [1, 2, 11–24]

For many years, heart failure pharmacotherapy primarily focused on neurohormonal modulation of the RAAS with angiotensin-converting enzyme inhibitors (ACEi), angiotensin receptor blockers (ARBs) and mineralocorticoid receptor blockers (MRAs), as well as the sympathetic nervous system with β blockers. However, advancements in the understanding of HF pathophysiology yielded new therapeutic targets, and drugs like SGLT2 inhibitors or angiotensin receptor neprilysin inhibitors (ARNI) have been developed and incorporated into management guidelines [5]. Recent data has demonstrated that up-titration and optimization of guideline directed medical therapy (GDMT) is crucial for HF patients as it improves quality of life and prognosis, although it is seldom easy to achieve in practice [6–10]. ARNI drugs in particular utilise a dual action on the two systems involved in heart failure pathophysiology and upregulate cardioprotective NPs in addition to RAAS blockade, translating into favourable outcomes especially in HFrEF [11]. This review will elucidate the mechanism of action of Sacubitril/Valsartan (Sac/Val) and outline the evidence for this drug across the spectrum of heart failure (Fig. 1).

### Mechanism of action

Sac/Val affects multiple systems and molecules in HF pathophysiology. Understanding its therapeutic effects in HF requires a review of the RAAS, NPS and neprilysin.

### Renin–angiotensin–aldosterone pathway

The renin–angiotensin–aldosterone system (RAAS) is a critical neurohormonal regulatory system affecting blood pressure, blood volume and maintaining tissue perfusion through downstream signaling molecules. Renin is secreted from juxtaglomerular cells, stimulated by several factors. Decreased renal blood flow detected by the afferent arteriole baroreceptor mechanism, reduced chloride ion delivery and beta-1 receptor activation all promote renin secretion [25]. Renin catalyses angiotensinogen conversion to angiotensin I, which is further converted to angiotensin II by angiotensin-converting enzyme (ACE). Angiotensin II further stimulates aldosterone secretion, which decreases natriuresis and diuresis through mineralocorticoid receptors in distal renal tubules and causes constriction of small arterioles [26].

Angiotensin II, acting via AT1 receptors, produces vasoconstriction by preventing the reuptake of noradrenaline in nerve terminals, enhances sodium reabsorption and promotes vascular and cardiac hypertrophy [27, 28]. AT2 receptors promote sodium excretion and diuresis [29]. Whilst normally, the proportion of AT2 to AT1 receptors in the heart is about 2:1, AT1 receptor expression increases in HF, leading to exaggerated vasoconstriction, aldosterone secretion, hypertrophy and catecholamine release [25].

Whilst in the initial stages of heart failure, the RAAS aids in maintaining homeostasis, its chronic activation is detrimental to the progression of the disease. Chronic excess of aldosterone and angiotensin II leads to endothelial and myocardial inflammation and fibrosis, persistent sympathetic stimulation and beta-adrenergic receptor downregulation, baroreceptor dysfunction, cardiac remodeling and the development of congestion [30, 31].

### Natriuretic peptides

The natriuretic peptide system (NPS) counters angiotensin II and aldosterone, reduces sympathetic tone and downregulates antidiuretic hormone. Its subtypes include atrial natriuretic peptide (ANP), brain natriuretic peptide (BNP) and C-type natriuretic peptide (CNP) [32]. ANP and BNP are found in myocardial tissue, with ANP primarily released in atria and BNP in ventricles, triggered by mechanical stretch of the myocardium due to high circulating volume (although other molecules like endothelin I, angiotensin II and catecholamines have been implied in their secretion) [33]. Specifically, proBNP is secreted by cardiomyocytes and cleaved into the active BNP and the inert NT-proBNP—both of which act as useful biomarkers in diagnosing HF, assessing treatment response and prognosis [34]. Acting on NPR-A, NPR-B and NPR-C transmembrane receptors [35], NPs cause venodilation and arterial dilation, decrease myocardial fibrosis and hypertrophy and lower the sensitivity of baro- and chemoreceptors, modulating compensatory responses in HF [33, 36]. In the nephron, they downregulate the RAAS and induce natriuresis and diuresis [33]. NP levels rise in HF, relieving congestion, increasing sympathetic tone and vasopressin secretion, but become less effective at improving cardiac function when upregulated chronically [37, 38].

### Neprilysin

NPs are broken down by neprilysin, an endopeptidase distributed widely across a range of tissues including the renal tubules, cardiomyocytes and endothelium. It is also responsible for breaking down several other vasoactive molecules, including vasodilatory (Substance P, bradykinin, adrenomedullin) and vasoconstrictive ones (angiotensin I and II, endothelin I, neurotensin) [39].

Substance P acts to increase cholinergic transmission and hence increase chronotropy and contractility [40]. Bradykinin produces potent vasodilation by increasing nitric oxide (NO), prostacyclin and endothelium-derived hyperpolarising factor (EDHF) levels [41]. Lastly, adrenomedullin causes potent, peripheral, NO-mediated vasodilation and might have a role in cardiac hypertrophy and fibrosis [42], stimulation of ANP secretion [43], as well as inhibition of thirst and salt craving [44, 45] and the RAAS [46, 47]. It also maintains the vascular endothelial barrier function and may oppose pulmonary and peripheral oedema in heart failure, since its levels rise proportionally to pulmonary congestion [48].

### Rationale for angiotensin receptor neprilysin inhibitors

Several attempts at targeting natriuretic peptides to derive therapeutic benefit were made before angiotensin receptor neprilysin inhibitors (ARNI). Exogenous natriuretic peptides were investigated [49], as well as neprilysin alone [50] and with ACEi [51], but neither showed benefit in trials. While neprilysin alone brought no significant benefit, combined with ACEi it caused harm due to the drug inhibiting both ACE and neprilysin, causing a build-up of bradykinin and angioedema. In 2015, Sac/Val, the first ARNI drug, was accepted to market by the FDA and ESA. It comprises a 1:1 combination of an angiotensin receptor blocker (valsartan) with a neprilysin inhibitor (sacubitril) [52].

Angiotensin receptor blockers inhibit downstream signalling mediated by the AT1 receptor. Its actions are, in principle, similar to ACEi [53]. Differences arise due to ARBs not influencing ACE levels and bradykinin breakdown, avoiding side effects like cough and angioedema [27]. ARBs cause vasodilation, reduce sympathetic tone and induce natriuresis and diuresis by blocking renal and neurohormonal effects of angiotensin II and aldosterone. They also inhibit cardiac and vascular remodelling [53].

**Fig. 2:**
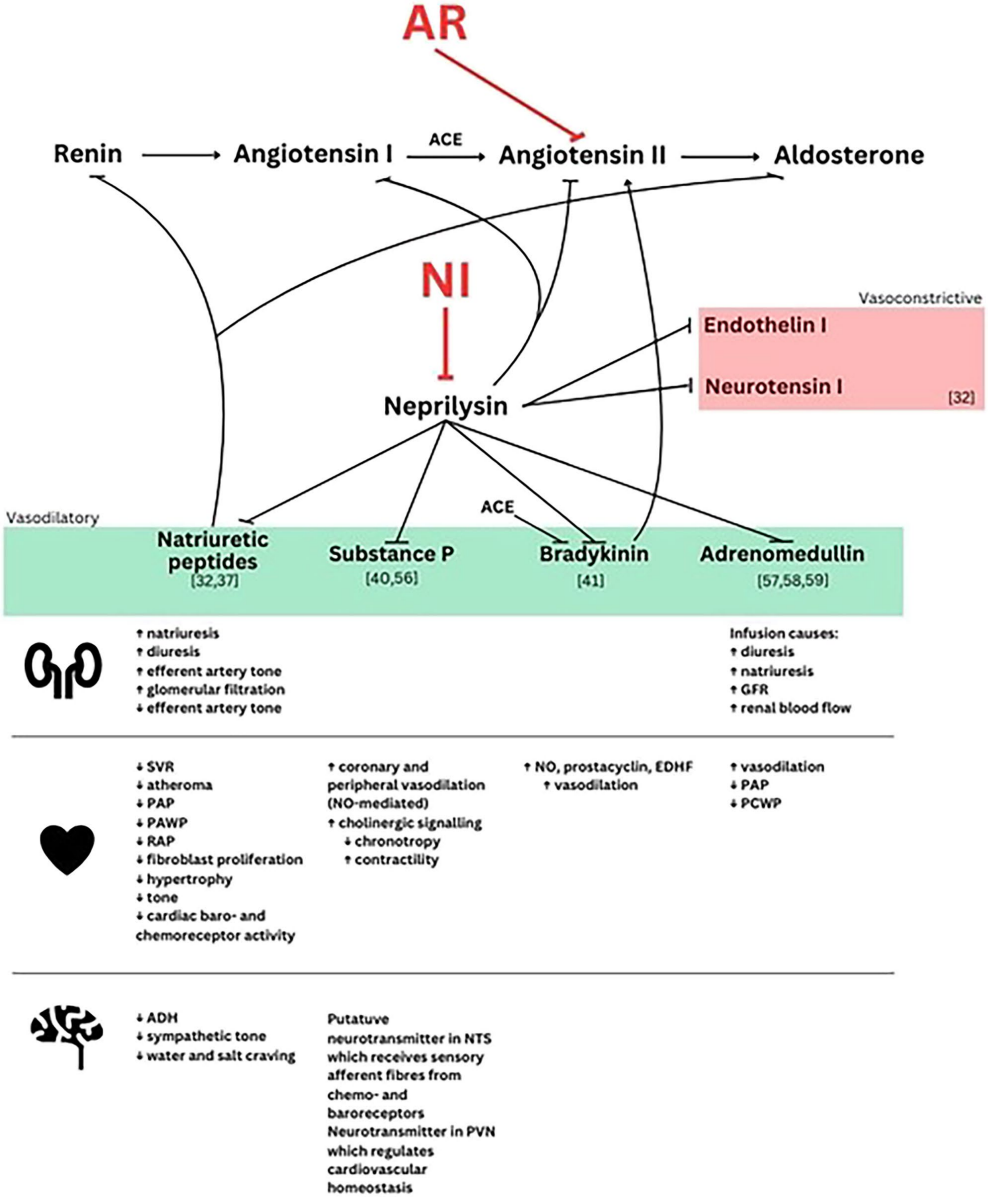
Mechanism of action of angiotensin receptor neprilysin inhibitors specific to heart failure and cardiovascular function [32, 37, 40, 41, 56–59]. *ACE* angiotensin-converting enzyme, *SVR* systemic vascular resistance, *PAP* pulmonary artery pressure, *PAWP* pulmonary artery wedge pressure, *RAP* right atrial pressure, *ADH* antidiuretic hormone, *NO* nitric oxide, *NTS* nucleus tractus solitarius, *PVN* paraventricular nucleus, *EDHF* endothelium-derived hyperpolarising factor, *GFR* glomerular filtration rate

Neprilysin inhibition leads to increased levels of its degradation substrates, potentiating the action of NPs, bradykinin, adrenomedullin and enkephalin [5]. Decreased breakdown of angiotensin I, angiotensin II and endothelin I however, constitutes an undesired effect in HF due to the vasoconstrictive, profibrotic effects of these molecules, similarly to decreased bradykinin breakdown and risk of angioedema [39, 54]. Combining the two classes aimed to resolve the undesired upregulation of angiotensin I, II and endothelin I. Figure 2 summarises the ARNI mechanisms of action.

### Cellular mechanisms of ARNI

Experimental evidence in animal models provides insight into the cellular mechanisms of ARNI. In various models of cardiomyocyte dysfunction, Sac/Val had elicited positive effects on cardiac remodelling, oxidative stress, apoptosis, fibrosis and inflammation [55]. These are detailed further in Table 1.

**Table 1:** Studies investigating molecular mechanisms of Sac/Val in various models of cardiomyocyte dysfunction in animals and human participants

Mechanism	Authors	Model	Effects of Sac/Val
Cardiac hypertrophy	[60]	Mouse model of MI via ligation of LAD	↓ ANP and β-MHC expression ↓ hypertrophy
	[61]	Rat HFpEF model induced via TAC	↓ Myh-7 expression ↓ LV weight ↓ hypertrophy
	[62]	TAC-induced HF in mice	↓ mRNA markers of ANP, BNP, β-MHC ↓ HW/BW ratio
	[62]	Phenylephrine-induced hypertrophy in neonatal rat cardiomyocytes	↑ Sirt3/MnSOD ↓ hypertrophy
Oxidative stress	[62]	TAC-induced HF in mice	↓ ROS ↓ apoptosis
	[63]	Cardiorenal syndrome induced by 5/6 nephrectomy and intraperitoneal doxorubicin	↑ NADPH oxidase 4 ↑ xanthine oxidase ↑ catalase ↓ ROS
	[64]	Isoprenaline-induced MI in rats	↓ superoxide dismutase ↓ catalase ↓ glutathione-S-transferase ↓ glutathione peroxidase ↓ glutathione reductase
Cardiac fibrosis	[65]	Rat model of MI via ligation of LAD	↓ Wnt/β catenin ↑ sFRP-1 ↓ fibrosis
	[66]	Rat model of MI via ligation of LAD	↓ TGF- β/SMADS activity ↓ fibrosis
	[62]	TAC-induced HF in mice	↓ collagen I, collagen III, TGF-β, and CTGF transcription ↓ *α*-SMA deposition ↓ fibrosis
Inflammation	[67]	HFrEF in human participants	↓ TNFα ↓ IL-18
	[68]	Apolipoprotein E deficient mice	↓ IL-6 ↓ MMP-8
			↓ MCP-1
Apoptosis	[69]	STZ-induced diabetic cardiomyopathy in rats	↓ AGEs ↓ CHOP ↓ PERK ↓ RAGE ↓ ER stress
	[70]	Doxorubicin-induced dilated cardiomyomathy in mice	↓ Drp1 ↓ Drp1 ser616 phosphorylation ↓ mitochondrial dysfunction and apoptosis

*AGEs* advanced glycation end products, *α-SMA* α smooth muscle actin, *β*-*MHC* β myosin heavy chain, *BW* body weight, *CHOP* C/EBP homologous protein, *CTGF* connective tissue growth factor, *ER* endoplasmic reticulum, *HW* heart weight, *ROS* reactive oxygen species, *MCP*-*1* monocyte chemotactic protein-1, *PERK* protein kinase R-like endoplasmic reticulum kinase, *ROS* reactive oxygen species, *RAGE* receptors for advanced glycation end products, *sFRP* secreted frizzled-related proteins, *TAC* transverse aortic constriction, *TGF β* transforming growth factor β

### Cardiac reverse remodeling and therapeutic benefit of Sac/Val

Cardiac reverse remodelling (CRR) refers to the reversal of abnormal atrial and ventricular volume, dimension and shape seen in HF [71]. Wang et al. [71] conducted a meta-analysis to compare the effect of ARNI vs. ACEi/ARB on cardiac reverse remodelling indices. They report an improvement of left ventricle (LV) size and hypertrophy in ARNI-treated HFrEF patients, compared to ACEi/ARB, even after short follow-up periods. Moon et al. [72] retrospectively analysed temporal patterns of echocardiographically-derived cardiac function parameters and occurrence of CVD death and hospitalisation in 415 patients treated with Sac/Val during a median follow-up of 19.1 months. In patients with no outcome events, EF, left ventricular end systolic/diastolic volume indices (LVEDVi, LVESVi), left atrial volume index (LAVi), mitral E/e′ ratio and pulmonary artery systolic pressure (PASP) significantly improved during 6 months post treatment initiation, in contrast to those with an outcome event in whom parameters did not improve. Overall, reverse remodelling occurred in 54.3% of patients at 6 months and 60.9% at 12 months. Other reports also found a beneficial impact of Sac/Val on echocardiographic indicators of reverse remodelling [73–77].

### Sac/Val and cardiac reverse remodelling surrogates (PROVE-HF trial)

To determine whether changes in NT-proBNP in patients treated with Sac/Val correlate with cardiac remodelling (indicated by LVESVi, LVEDVi, LVEF and LAVi), the PROVE-HF trial [78] was conducted. A 12-month, open-label trial, it enrolled 794 patients with HFrEF who were initiated on Sac/Val with repeated echocardiographic and NP monitoring. At 12 months, significant correlations were seen between changes in log_2_-NT-proBNP and LVEF, LVEDVi, LVESVi, LAVi and E/e′. This suggests a similar pattern of reverse myocardial remodelling associated with reduction of NT-proBNP seen in other GDMT [79].

### Sac/Val and haemodynamic parameters in HF

Heart failure is inevitably associated with haemodynamic changes like reduced cardiac output, raised LV filling pressures or pulmonary congestion [80]. Increased filling pressures are associated with adverse cardiac events and cardiovascular mortality [81], thus it is important to consider haemodynamic impacts of Sac/Val therapy. In one prospective observational study, Fröhlich et al. [82] followed 37 outpatients with HFrEF, obtaining haemodynamic parameters non-invasively by use of inert gas rebreathing and bioimpedance cardiography at baseline, at 2 weeks, 3 months and 6 months post-initiation of Sac/Val. ARNI was associated with improvements in LVEF, PAP and NT-proBNP. Initially, a decline in systemic vascular resistance index (SVRi) and systolic blood pressure (SBP) were mirrored by a decrease in stroke volume (SV) and cardiac index (CI), but the latter two parameters steadily recovered and surpassed baseline levels, potentially reflecting reverse remodelling induced by Sac/Val. Zhang et al. [83] conducted a meta-analysis of 10 trials involving 875 patients with HFrEF, where right ventricular and pulmonary pressure parameters were reported after Sac/Val initiation, noting significant improvements in tricuspid annular peak systolic velocity, tricuspid annular peak systolic velocity and pulmonary artery systolic pressure (PASP). Gentile et al. [84] found a similar haemodynamic shift (assessed invasively) in 53 patients with advanced HF, noting a significant decline in PASP and mPAP after Sac/Val initiation.

Brockmöller et al. [85] investigated haemodynamic changes after Sac/Val initiation in 53 patients with HFpEF and isolated postcapillary hypertension (IpcPH), as well as 13 patients with combined pre- and postcapillary hypertension (CpcPH). They report significant declines in mPAP, PASP and PADP in both patient groups, whereas the PCWP decreased significantly only in the CpcPH group, with only a tendential decrease in the IpcPH group. Mean right atrial (RA) pressures were lower in the IpcPH group after Sac/Val initiation. No differences in other invasively measured parameters like CO, CI or SVR were seen in either group. The recent PARABLE (Personalized Prospective Comparison of ARNI With ARB in Patients With Natriuretic Peptide Elevation) trial explored whether Sac/Val would improve markers of cardiac structure and function in 250 asymptomatic, pre-HFpEF patients compared with valsartan. While LAVi and LVEDVi both showed significant improvement in the Sac/Val arm, E/e′ showed no differences between treatment groups, with both showing significant reduction.

## Clinical outcomes of Sac/Val in HFrEF

### Prospective comparison of ARNI with ACEI to determine impact on global mortality and morbidity in heart failure (PARADIGM-HF trial)

In 2014, the PARADIGM trial of ARNI in HFrEF was the first to show significant benefit of sacubitril-valsartan compared to enalapril [11]. The randomised, double-blind trial recruited patients with New York Heart Association (NYHA) class II-IV HF, with an EF ≤ 40%. In patients receiving Sac/Val, the primary outcome (composite of death from CVD or HF hospitalisation) occurred in 21.8% vs. 26.5% in the enalapril group (HR 0.8; 95% CI 0.73 to 0.87; *P* < 0.001). Death from CVD, all-cause mortality and HF hospitalisation were significantly reduced in the ARNI group (*P* < 0.001), with the effect apparent across the EF spectrum studied [86]. The Sac/Val arm also showed significantly lower median NT-proBNP levels at one month compared with the enalapril arm, falling ≤ 1000 pg/mL in 31% vs. 17% of enalapril-treated patients [87]. Incidence of the primary endpoint was 59% lower in patients with such a decline. Less patients on Sac/Val withdrew due to adverse effects, and lower rates of cough and hyperkalemia were observed in this group [11]. The ARNI group did, however, show symptoms of hypotension and non-serious angioedema more frequently. This and other key trials are summarised in Table 2.

**Table 2:** Summary of key clinical trials of Sac/Val in HFrEF and HFpEF

	Trial, (*n*)	Enrolment criteria	Primary outcome	Comparators	Results
HFrEF	PARADIGM-HF [11] (*n* = 8442)	LVEF ≤ 40% NYHA II-IV ↑ NT-proBNP/BNP	Death from CVD or HF hospitalisation	Sac/Val vs. enalapril	Primary outcome occurred in 21.8% of patients in Sac/Val group vs. 26.5% in enalapril (HR 0.8; 95% CI 0.73 to 0.87; *P* < 0.001)
	PIONEER-HF [1] (*n* = 736)	LVEF ≤ 40% Admitted 24 h–10 days prior with ADHF ↑ NT-proBNP/BNP SBP ≥ 100 mmHg	Change in NT-proBNP	Sac/Val vs. enalapril	46.7% reduction in NT-proBNP with Sac/Val at 8 weeks, compared to 25.3% in enalapril group (ROC 0.71; 95% CI 0.63–0.81; *P* < 0.001)
	LIFE trial [17] (*n* = 335)	LVEF ≤ 35% NYHA class IV ≥ 3 months of guideline-directed therapy ↑ NT-proBNP	AUC of NT-proBNP compared to baseline	Sac/Val vs. valsartan	No difference in AUC of NT-proBNP between Sac/Val and valsartan groups
	TRANSITION [98] (*n* = 1002)	LVEF ≤ 40% NYHA II-IV SBP ≥ 100 mmHg Hospitalised for ADHF	Proportion of patients reaching target dose at 10 weeks (97/103 mg BD)	Patients stratified according to RAAS use pre-admission, randomised to start Sac/Val ≥ 12 h from discharge or 1–14 days post-discharge	No difference between groups in proportion attaining max dose after 10 weeks (45.4% in pre-discharge initiation vs. 50.7% in post-discharge initiation; RR 0.90; 95% CI 0.79–1.02; *P* = 0.099)
	PROVE-HF [78] (*n* = 794)	LVEF ≤ 40% NYHA II–IV Stable dose of loop diuretic during last 2 weeks	Correlation between changes in log_2_–NT-proBNP and LVEF, LVEDVi, LVESVi, LAVi and E/e’ ratio at 12 months	Single group, open-label study	Change in log_2_–NT-proBNP over 12 months correlated with LVEF (*r* = − 0.381), LVEDVi (*r* = 0.320), LVESVi (*r* = 0.405), LAVi (*r* = 0.263) and E/e’ ratio (*r* = 0.269)
	PARADISE-MI [15] (*n* = 5661)	LVEF ≤ 40% Acute MI Pulmonary congestion One of eight prespecified risk factors	CV death, outpatient symptomatic HF or HF hospitalisation	Sac/Val vs. ramipril	No difference in incidence of primary event between groups
	TITRATION [107] (*n* = 498)	LVEF ≤ 35% NYHA II–IV Assessment of safety and tolerability of uptitration in the real-world	Safety, tolerability and success of uptitration during a 12-week period	Condensed regimen of uptitration (100 mg BD for 2 weeks, then 200 mg BD) vs. conservative regimen (50 mg BD for 2 weeks, 100 mg BD for 3 weeks, then 200 mg BD)	76% of patients achieved and maintained 200 mg BD dose after 12 weeks (77.8% vs. 84.3% for ‘condensed’ vs. ‘conservative’; *P* = 0.078). No difference in incidence of hypotension, hyperkalaemia, angioedema or renal dysfunction between the regimens
HFpEF	PARAGON-HF [108] (*n* = 4822)	≥ 50 years LVEF ≥ 45% NYHA II–IV ↑ NT-proBNP/BNP Evidence of structural heart disease Diuretic therapy	Total hospitalisations for HF and CV death	Sac/Val vs. valsartan	894 primary events in Sac/Val group and 1009 in valsartan group (RR 0.87; 95% CI 0.75–1.01; *P* = 0.06) Incidence of CV death 8.5% in Sac/Val, 8.9% in valsartan group (HR 0.95; 95% CI 0.79–1.16) Suggestion of heterogeneity between the subgroups—potential benefit of Sac/Val in those with lower LVEF and in women
	PARALLAX [14] (*n* = 2572)	≥ 45 years LVEF ≥ 40% NYHA II–IV KCCQ < 75	Change in NT-proBNP from baseline to week 12 Change in 6MWD from baseline to week 24	Sac/Val vs. background medication-based individualised comparators (enalapril, valsartan or placebo)	At 12 weeks, patients in Sac/Val group had a significantly greater reduction in NT-proBNP compared with comparator group, with an adjusted geometric mean ratio of 0.84 (95% CI 0.80–0.88; *P* < 0.001) No significant differences in change in 6MWD at week 24 between the two groups
	PARAGLIDE-HF [18] (*n* = 466)	LVEF > 40% ↑ NT-proBNP/BNPCurrently hospitalized for WHF / within 30 days of WHF episode	Time-averaged, proportional change in NT-proBNP at weeks 4 and 8 compared to baseline	Sac/Val vs. valsartan	Time-averaged reduction in the NT-proBNP was greater with Sac/Val (ROC 0.85; 95% CI 0.73–0.999; *P* = 0.049) Evidence of larger treatment effect in subgroup with LVEF ≤ 60%

A post-hoc analysis of the PARADIGM trial aimed to investigate clinical progression of HF in surviving patients [88]. It found that Sac/Val was associated with a ~ 16% lower incidence of clinical deterioration (indicated by addition of a new drug, intravenous therapy use or an increase in diuretic dose for over a month) and a lower incidence of emergency department visits (HR 0.66; 95% CI 0.52–0.85; *P* = 0.001).

### LCZ696 in advanced heart failure (LIFE trial)

Although enrolment criteria for the PARADIGM trial included patients with NYHA classes II-IV, 99% of patients had NYHA class II-III HF [17]. Due to limited evidence and clinical experience of Sac/Val in advanced HFrEF, the LIFE trial compared treatment with Sac/Val and valsartan alone in this patient population [17]. This randomised, double-blind trial enrolled 335 patients with NYHA class IV heart failure with EF ≤ 35%, raised NPs and at least one other clinical indicator of advanced HF. The primary endpoint was the AUC of NT-proBNP compared with baseline at 2, 4, 8, 12 and 24 weeks. Although in both the valsartan and Sac/Val groups, NT-proBNP levels declined below baseline at 8 weeks, no differences in AUC for NT-proBNP between the two groups were noted.

## Clinical outcomes of Sac/Val in HFpEF

### The effect of sacubitril/valsartan on left ventricular myocardial deformation in heart failure with preserved ejection fraction (PARAMOUNT trial)

The PARAMOUNT trial was the first randomised, double-blind trial to assess the safety and efficacy of ARNI in HFpEF [89]. It enrolled patients with NYHA Class II-IV, EF ≥ 45% and an NT-proBNP > 400 pg/mL. Compared to participants receiving valsartan alone, the ARNI group saw a significant reduction in NT-proBNP at 12 weeks, which was the primary endpoint of the trial (HR 0.77, 95% CI 0.64–0.92, *P* = 0.005). The ARNI group showed reversed left atrial remodelling, reduced left atrial size and a downgrade in NYHA class at 36 weeks. It also showed a safety profile alike valsartan alone, with 15% of patients reporting adverse effects in the ARNI group vs. 20% in the valsartan group. Subsequent analysis of high-sensitivity troponin T revealed high hs-TnT in 55% of patients at week 0, which was significantly reduced in the ARNI group compared with valsartan at week 12 (*P* = 0.05) and 36 (*P* = 0.03) [90]. The improvement in the above parameters suggested that ARNI might reduce the extent of myocardial injury in HFpEF.

### Prospective comparison of ARNI with ARB on global outcomes in HF with preserved ejection fraction (PARAGON-HF trial)

Several years later, the PARAGON trial investigated whether results from PARAMOUNT translate into improved clinical outcomes [13]. It recruited patients with NYHA II-IV, an EF ≥ 45%, raised NPs and structural heart disease. No significant differences in primary outcome (composite of death due to CVD or HF hospitalisation) were observed between the two trial groups (HR 0.87; 95% CI 0.75 to 1.01; *P* = 0.06). Primary outcome component analysis showed an improvement in NYHA class (odds ratio 1.45; 95% CI 1.13–1.86) and a lower incidence of deterioration of kidney function (HR 0.50; 95% CI 0.33–0.77) in the Sac/Val group. No significant changes in the Kansas City Cardiomyopathy Questionnaire (KCCQ) score were observed. The pattern of adverse effects followed that reported in PARADIGM-HF, with more hypotension and angioedema in the ARNI group. Subgroup analysis revealed a possible heterogeneity in treatment effect in subgroups with lower ejection fractions and in women, both of which were suggested to benefit from treatment with Sac/Val.

Cunningham et al. [91] evaluated whether the baseline or change in NT-proBNP affected clinical outcomes in patients enrolled into PARAGON, if it modified response to Sac/Val treatment, and if Sac/Val affected NT-proBNP. They found a consistent decline in NT-proBNP in the Sac/Val group, evident across the LVEF spectrum in both sexes. Baseline NT-proBNP had a strong association with subsequent HF hospitalisation and CV mortality, but did not modify Sac/Val effects. Those with larger reductions in NT-proBNP had better clinical outcomes.

**Fig. 3:**
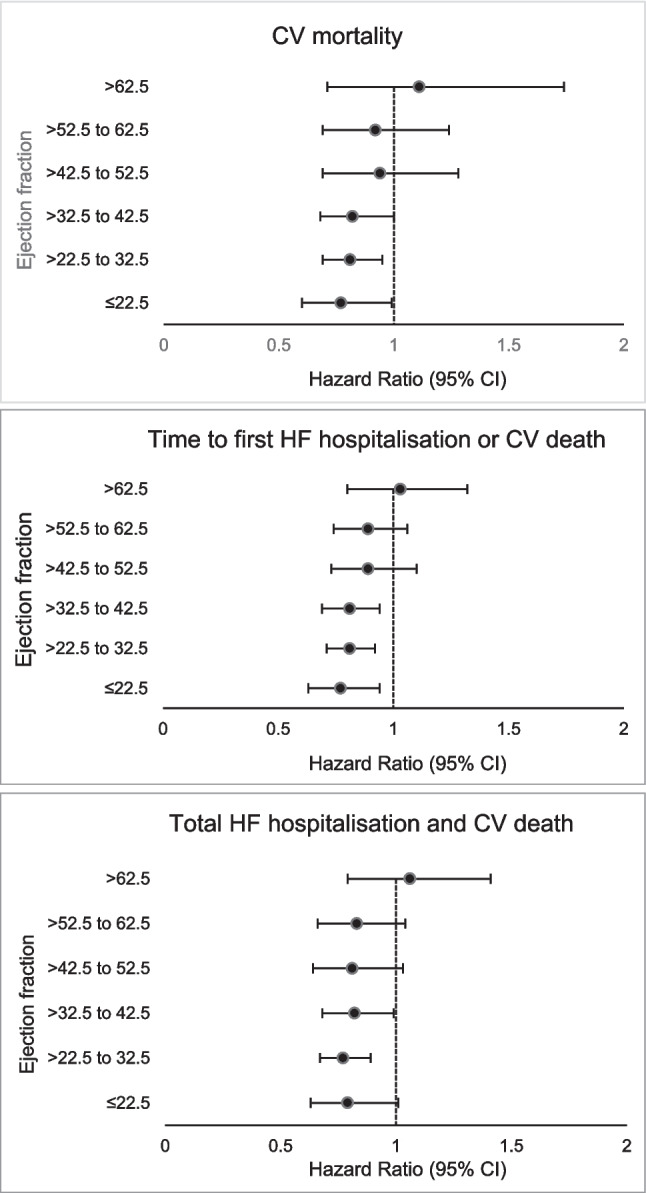
**a**–**c** Effect of Sac/Val compared to active comparator on **a** CV mortality, **b** time to first HF hospitalisation and **c** total HF hospitalisation and CV death. Data is divided by patients’ EF range at baseline[1, 92]

**Fig. 4:**
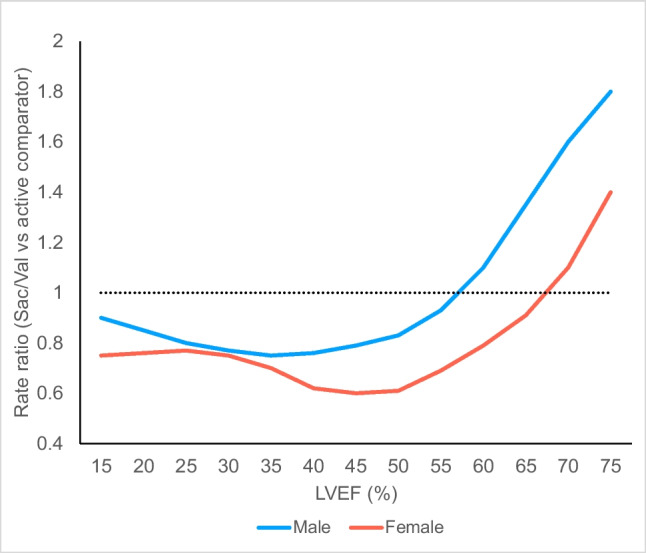
Continuous treatment effect of Sac/Val compared to active comparator (valsartan or enalapril) by LVEF and gender. Rate ratio for the composite of heart failure hospitalisation and CV death is shown. Graph adapted from Solomon et al. [92]

Pooled data from PARAGON and PARADIGM confirmed that treatment benefits of Sac/Val varied by EF (treatment-by-continuous LVEF interaction *P* = 0.02), with mostly patients with EF below 57% benefitting [92] (Fig. 3a–c). EF thresholds for therapeutic benefit also varied by sex, with men seeing benefit across a narrower range of LVEF and females seeing benefit extending up to higher LVEF (RR Upper CI = 1.0 at 45% vs. 60%) (Fig. 4). Even at ejection fractions below these thresholds, Sac/Val reduced HF hospitalisation and incidence of primary outcome significantly more in women [93]. In response to this meta-analysis and subgroup results of the PARAGON trial showing a reduction in the incidence of HF hospitalisation in patients with an LVEF < 57%, the FDA had endorsed the use of Sac/Val in patients with an LVEF “less than normal” [94], while the European Society of Cardiology (ESC) made a Class IIb recommendation that Sac/Val may be considered in HF with mildly reduced EF (HFmrEF) [95].

### Prospective comparison of ARNI vs. comorbidity-associated conventional therapy on quality of life and exercise capacity (PARALLAX trial)

The PARALLAX trial evaluated Sac/Val vs. GDMT in terms of the change in NT-proBNP at 12 weeks and measures of exercise capacity at 24 weeks [14]. This was a randomised, double-blind trial enrolling 2572 patients ≥ 45 years old, with symptomatic heart failure requiring diuretics and showing elevated NT-proBNP. Similarly to previous trials, NT-proBNP significantly declined in the Sac/Val group compared to the comparator at 12 weeks (adjusted geometric mean ratio of 0.84; 95% CI 0.80–0.88; *P* < 0.001). The 6MWD, KCCQ score and NYHA class showed no significant differences between groups, although sex modified the effect on 6MWD with women showing an improvement.

## Acute decompensated HF and Sac/Val

### Comparison of sacubitril/valsartan versus enalapril on effect on NT-proBNP in patients stabilized from an acute heart failure episode (PIONEER-HF trial)

PIONEER was the first large-scale, double blind, active-controlled trial examining Sac/Val after haemodynamic stabilisation in acute decompensated heart failure (ADHF) [1]. It enrolled 887 patients with an LVEF ≤ 40%, NT-proBNP ≥ 1600 pg/mL or a BNP ≥ 400 pg/mL, currently hospitalised and admitted with a primary diagnosis of ADHF 24 h–10 days prior to enrolment. Patients were randomised to Sac/Val at a target dose of 200 mg BD or enalapril 10 mg BD, and followed up every 2 weeks throughout the 8-week trial period. The time-averaged change in NT-proBNP (the primary endpoint) was significantly greater in patients on Sac/Val (percent change, − 46.7% vs. − 25.3%; ROC 0.71; 95% CI 0.63–0.81; *P* < 0.001), which was evident from week 1 onward. High-sensitivity troponin T levels also followed this pattern, showing a significant decline in the Sac/Val group. An analysis of an exploratory composite endpoint of HF rehospitalisation and CV death showed reduced incidence in Sac/Val [96]. Interestingly, the safety profile was similar in both groups. This data provides reassurance about the safety and efficacy of Sac/Val in the vulnerable period after hospitalisation for ADHF and supports its use in black patients (35.9% of those enrolled identified as black) with equal reductions in NT-proBNP in this population and no cases of angioedema.

### Changes in NT-proBNP, safety, and tolerability in HFpEF patients with a WHF event (HFpEF decompensation) who have been stabilized and initiated at the time of or within 30 days post-decompensation (PARAGLIDE-HF)

A post-hoc analysis of PARAGON showed a greater absolute risk reduction of primary outcome with Sac/Val in patients enrolled early after hospitalisation (6.4% if ≤ 30 days from hospitalisation, 4.6% if 31 to 90 days, 3.4% if 91 to 180 days) [97]. The recent PARAGLIDE-HF trial gave further insight into the effectiveness and safety of Sac/Val in HFpEF patients with decompensation [18]. This was a double-blind, randomised, controlled trial of Sac/Val vs. valsartan in patients with an EF > 40% with an event of HF worsening within the past 30 days. The primary outcome was the time-averaged, proportional change in NT-proBNP at weeks 4 and 8 compared to baseline, with a secondary hierarchical outcome of CV death, HF hospitalisation, urgent HF visits and change in NT-proBNP. The time-averaged reduction in NT-proBNP was greater with Sac/Val (ROC 0.85; 95% CI 0.73–0.999; *P* = 0.049). As with previous trials, there was evidence of a larger treatment effect in those with an EF ≤ 60% in terms of NT-proBNP change and secondary outcome incidence.

Morrow et al. [12] analysed pooled data from PIONEER and PARAGLIDE (*n* = 1130), with the aim of evaluating the safety and efficacy of Sac/Val across the LVEF spectrum in ADHF. They report a 24% greater reduction in NT-proBNP with Sac/Val vs. control (ROC 0.76; 95% CI 0.69–0.83; *P* < 0.0001), and a 30% reduction in CV death or hospitalisation for HF with Sac/Val vs. control (HR 0.70; 95% CI 0.54–0.91; *P* = 0.0077). This reduction was seen in patients with a baseline LVEF < 60%. In terms of adverse effects, hypotension was expectedly more frequent in the Sac/Val group.

### TRANSITION trial

The TRANSITION trial was a randomised, open-label trial which aimed to investigate the optimal time of Sac/Val initiation post-ADHF episode [98]. Patients hospitalised for ADHF were randomised to two treatment initiation regimes—either receiving Sac/Val ≥ 12 h before discharge or on days 1–14 after discharge. The trial’s primary endpoint was the successful up-titration to 200 mg twice daily after 10 weeks. Results show no significant differences in the proportion of patients in pre- vs. post-discharge initiation groups that met the primary endpoint (45.4% vs. 50.7% respectively; RR 0.90; 95% CI 0.79–1.02; *P* = 0.099). Age < 65, SBP ≥ 120, history of hypertension, no AF, eGFR ≥ 60 mL/min/1.73 m^2^ and de novo HF were all significant predictors of attaining the maximal dose of 200 mg twice daily at 10 weeks, whilst time of initiation and previous RAAS inhibitor exposure were not. Authors attribute the lower proportion of patients that were successfully up-titrated to 200 mg at week 10 compared to PIONEER to the patient population in that trial being younger, having less comorbidities and more de novo HF, all of which have been identified as predictors of successful up-titration. This trial, along with PIONEER, show the feasibility of Sac/Val in patients after ADHF, both when initiated prior to, or after discharge. It also shows a discrepancy in the proportion of patients who were able to achieve doses of 100–200 mg in the trial setting vs. in clinical practice, with 62% of patients reaching that dose at week 10 vs. only 43% on a dose higher than 50 mg among outpatients in Germany [99].

## Sac/Val post-acute myocardial infarction (AMI)

### Prospective ARNI vs. ACE inhibitor trial to determine superiority in reducing heart failure events after myocardial infarction (PARADISE-MI)

The PARADISE-MI trial evaluated the safety and efficacy of Sac/Val used in 5661 patients with AMI with concurrent reduced LVEF ≤ 40%, pulmonary congestion and at least one of eight prespecified risk factors [15]. Patients were randomly assigned to receive either Sac/Val or ramipril, and previous ACEi and ARBs were discontinued at randomisation. The primary outcome was CV death, outpatient symptomatic HF or HF hospitalisation. No significant differences in the incidence of the primary outcome were noted between trial arms, including in exploratory analyses of endpoint components. Although the trial had sufficient power to detect the anticipated treatment effect size, authors noted the vastly lower mortality rates in this trial compared to previous MI trials involving ACE inhibitors [39]. A subsequent post hoc analysis involving all investigator-reported events showed a significantly lower incidence of events in the Sac/Val arm (RR 0.79; 95% CI 0.67–0.93; *P* = 0.004) [100]. As noted by Bellis et al. [101], the STEMI subgroup did show a trend towards superiority of Sac/Val over enalapril, and this resembled the results of a meta-analysis of trials enrolling STEMI patients exclusively which found a possible benefit of early Sac/Val (within 24 h post-PCI) compared to ACEi/ARB [102]. Mechanistically, the addition of a neprilysin inhibitor could provide additional benefit due to neprilysin playing a greater role in bradykinin regulation at higher bradykinin levels (as is the case in AMI), and the activation of the PI3K-Akt/GSK-3β cardioprotective pathway by molecules normally inhibited by neprilysin [101].

Liu et al. [77] enrolled 275 patients with HFrEF after ACS with PCI, treated with Sac/Val or routine, guideline-directed therapy. The symptoms, NT-proBNP, LVEF, left ventricular mass index (LVMI), LVEDVi, LVESVi, major adverse cardiac events (MACEs) and adverse reactions, were recorded at baseline and at 6 months follow up. The Sac/Val group had significantly greater improvements in LVEF (*P* = 0.006), NT-proBNP (*P* = 0.009), LVEDVi (*P* = 0.001) and LVESVi (*P* = 0.001) compared to the group receiving guideline-directed medical therapy (GDMT). A significant improvement in systolic function with Sac/Val after STEMI was also reported by Rezq, Saad and el Nozahi [103]. Interestingly, subgroup analysis showed that the incidence of MACE was significantly higher in patients with unstable angina compared to NSTEMI, potentially indicating Sac/Val’s superior efficacy in MI due to its neprilysin-driven effects (summarised by Bellis [101]) on limiting reperfusion injury. Given these results, it also remains uncertain why improved cardiac function did not translate into clinical outcomes.

Contradictory results were reported by Shah et al. [104] from the echo substudy of the PARADISE trial, which followed 544 patients from the trial. The primary endpoint was LVEF and LAV change, and secondary endpoints included changes in LVEDV and LVESV at 8 months. No significant differences in the primary endpoint were found between Sac/Val and ramipril groups, although Sac/Val was associated with less increase in LVEDV (*P* = 0.025) and greater decline in LV mass index (*P* = 0.037), increase in tissue Doppler e’lat (*P* = 0.005), decrease in E/e’lat (*P* = 0.045) and decrease in tricuspid regurgitation peak velocity (*P* = 0.024). This pattern of changes, that has also been reported in PRIME and EVALUATE-HF trials [105, 106], is more consistent with Sac/Val’s effect on filling pressures instead of systolic function.

### Dose up-titration and adherence

A big discrepancy exists between the recommended target dose of Sac/Val and actual prescriptions in outpatients, with 43% achieving a dose higher than 50 mg in real-life scenarios (Germany) [99] and 30% on the target dose in a multinational observational study [8, 10, 109]. In rigidly controlled trials like TRANSITION, target doses were achieved in 62% of patients at 12-week follow up, highlighting that up-titration in feasible in most. TITRATION—a RCT assessing the tolerability of Sac/Val uptitration from 50 to 200 mg twice daily over 3 and 6 weeks in HFrEF patients, found that 76% tolerated up—titration to the target dose [107]. Previous studies emphasise the importance of optimal Sac/Val treatment to derive most clinical benefit. A PARADIGM post-hoc analysis shows that dose reduction of Sac/Val is associated with an increase in primary outcome incidence [110], and Martens et al. [111] reported a dose-dependent improvement in LVEF and LVESV.

Adherence is an important patient-related barrier to optimal guideline-directed therapy [8, 112]. Carnicelli et al. [113] evaluated adherence to Sac/Val post-discharge in patients with HFrEF by analysing the timeframes of medication refills and calculating the proportion of days covered (PDC). They found that 67.1% of the 897 patients had a PDC < 80% during the 3 months post-discharge. Patients with a PDC ≥ 80% (32.9%) had a significantly lower adjusted hazard of all-cause rehospitalisation (HR 0.66; 95% CI 0.48–0.89) and death (HR 0.42; 95% CI 0.22–0.79) at 3 and 12 months, and every 5% decrease in PDC was associated with a significant increase in rehospitalisation and death. A recent observational study provided insight into the real-world use of Sac/Val [114]. Adherence data for 387 patients showed complete adherence in 53%. In comparison, another study reported an adherence rate to ACE-Is/ARBs, beta-blockers, and MRAs to be 46.5%, 59.4%, and 35.6%, respectively [115].

## Safety profile of Sac/Val

### Hypotension

Several adverse effects are prominent with Sac/Val. Firstly, it reduces the breakdown of several vasodilatory molecules causing hypotension—the most reported adverse effect in real-life and in trials enrolling HFrEF and HFpEF patients [107, 116, 117].

In HFpEF, treatment-related hypotension is associated with an increased risk of HF hospitalisation, CV and all-cause mortality (adjusted HR 1.62; 95% CI 1.28–2.05; *P* < 0.001) [118]. LVEF modifies the association between Sac/Val treatment and hypotension, with patients with an LVEF > 60% at significantly higher risk.

In HFrEF, hypotension is an adverse effect significantly more prevalent in Sac/Val vs. enalapril-treated patients [119]. It is also the biggest barrier to uptitration and cause for discontinuation of Sac/Val. It is important to note, however, that the benefits of Sac/Val were consistent regardless of baseline BP in PARADIGM, including those with low BP [120]. Although mortality was higher in those with low BP, this is likely because it is overall a factor identifying HF patients at higher risk. A post-hoc analysis of PARADIGM analysed time-updated BP measurements (at time-points closest to an event or end of the trial) and BP changes over time, reinforcing Sac/Val’s efficacy in those with systolic BP < 110 mmHg during treatment [121]. Another post-hoc analysis of PARALLEL-HF also found that, although hypotension-related adverse effects were more common in the Sac/Val arm vs. enalapril, reduction in the risk of CV death or hospitalisation were similar in both groups in patients with or without hypotension [122].

### Hyperkalaemia

Hyperkalaemia is the second most reported adverse effect although, notably, its incidence is lower compared with enalapril [123]. This discrepancy might arise from Sac/Val not only eliciting a direct inhibitory effect on the RAAS via valsartan (and hence a lesser excretion of potassium through apical ROMK channels due to reduced aldosterone), but also a potent inhibitory effect of natriuretic peptides on aldosterone release. A secondary analysis of PARADIGM-HF showed that patients receiving Sac/Val alongside an MRA are less prone to develop hyperkalaemia compared with MRA and enalapril [124], supporting the exchange of ACEi for Sac/Val in HFrEF to elicit safe, concurrent use of MRAs.

### Worsening renal function

Worsening renal function is another recognised adverse effect of RAAS inhibiting drugs which, by definition, counteract compensatory mechanisms restoring renal blood flow. Although worsening renal function is one of the most reported adverse effects in Sac/Val, it is still relatively rare, reported in < 10% of patients [125]. The PARADIGM trial showed a similar incidence of new AKI or end-stage renal failure in both the Sac/Val and enalapril groups, although a subsequent post-hoc analysis revealed a slower decline in eGFR in patients on Sac/Val vs. enalapril (1.61 mL/min per 1.73 m^2^ compared with 2.04 mL/min per 1.73 m^2^; *P* < 0.001) [126]. Patients on Sac/Val also had a lower incidence of reaching a creatinine of 2.5 mg/dL and discontinuing ARNI due to renal events [127]. These results should be differentiated from the early, transient rise in eGFR post-initiation of Sac/Val, which is a common feature of ARNI therapy and has a neutral long-term impact [128]. The exact mechanisms of early eGFR dip related to the initiation of RASi is not completely understood, but from a clinical standpoint, it is crucial to understand that the prognostic significance of the phenomenon is very different from AKI [129].

A similar trend was seen in HFpEF, with comparable rates of AKI and worsening renal function in Sac/Val and candesartan seen in PARAMOUNT [89], but a lesser rate of eGFR decline in Sac/Val (1.5 mL/min per 1.73 m^2^ compared to 5.2 mL/min per 1.73 m^2^; *P* = 0.002) [130]. In PARAGON-HF, the risk of renal composite outcome (death from renal failure, end-stage renal disease, eGFR decline ≥ 50% from baseline) was decreased by 50% in the Sac/Val group [131].

### Emerging evidence of Sac/Val use

Several studies investigated the use of Sac/Val in new patient populations. Arcudi et al. [132] conducted a small study investigating its effects on LVEF in Duchenne Muscular Dystrophy (DMD), a condition highly associated with dilated cardiomyopathy. It followed 22 patients with DMD, including 6 with an EF < 40% and NYHA II-III, and 16 with EF > 40% and NYHA I-II. The treatment group (EF < 40%) was given a maximum tolerated dose of Sac/Val ± β blocker, while the control group (EF > 40%) received guideline-directed therapy, i.e. an ACEi ± β blocker. EF was measured at baseline and at follow up (median 7 months). All patients receiving Sac/Val showed improvement in EF at follow up (31% at baseline to 38% at follow up; *P* < 0.05), with no associated adverse effects. The control group had no significant change in EF. The difference in the mean of EF variation between groups was significant (*P* < 0.05). This suggests a role of Sac/Val in patients with Duchenne Muscular Dystrophy with dilated cardiomyopathy and reduced EF, although larger trials are needed to investigate whether EF changes translate into improved outcomes.

Cardiotoxicity and cardiac dysfunction are recognised adverse effects of breast cancer chemotherapy. Their occurrence is associated with increased morbidity, mortality and cessation of chemotherapy [133]. The MAINSTREAM trial aims to investigate whether Sac/Val provides benefit in preventing chemotherapy-related cardiotoxicity. It will be a randomised, placebo-controlled, double-blind, multicentre trial which will recruit 480 patients with early breast cancer, treated with anthracyclines and/or anti-HEGF2 randomised to Sac/Val or placebo. Patients will be followed-up for 24 months with echocardiography and biomarkers measured. The primary endpoint will be a decrease in LVEF by ≥ 5% within 24 months, with a secondary composite endpoint of all-cause mortality or hospitalisation for heart failure, as well as other imaging, laboratory and clinical outcomes. Results are expected in December 2027.

## Summary

As demonstrated in this review, Sac/Val is a useful therapeutic avenue in select patients with heart failure. International guidelines support its use in patients with HF with less-than-normal ejection fraction where it induces reverse cardiac remodelling and improves clinical outcomes including a beneficial effect on worsening renal function, coexisting diabetes mellitus and functional capacity. Its benefit has been shown both in acute and chronic contexts of heart failure. In other patient subgroups, like those with higher ejection fractions, those with NYHA Class IV symptoms or those post-MI, the benefit is less clear. Sac/Val is well-tolerated, although uptitration to target doses in real-life practice lacks behind the percentages achieved in trials. Adverse effects are comparable to other guideline directed therapies, with the exception of symptomatic hypotension which is more commonly reported with Sac/Val, especially in patients with higher ejection fractions.

Further research is needed to fill several gaps in knowledge. Identifying patient clinical phenotypes that would yield the greatest benefit from Sac/Val treatment is one such topic. The lack of efficacy of Sac/Val in the LIFE trial for example, might be attributable to the desensitisation of natriuretic peptide receptors and high BNP levels causing inhibition of neprilysin, making patients enrolled in the trial less responsive to Sac/Val therapy [39] (although the evidence base for Sac/Val in advanced HF remains scarce, with few NYHA IV patients enrolled in other clinical trials). Multiple pathophysiological pathways are also at play, causing decreased exercise tolerance in heart failure, and identifying phenotypic features that could be targeted with Sac/Val is necessary [14]. Metabolic effects of Sac/Val are also inadequately understood. Neprilysin causes hydrolysis of molecules that participate in glucose homeostasis and feeding behaviour, like GLP-1. Animal studies show that mice deficient in neprilysin experience an increase in body fat and dysregulation of lipid metabolism [39]. Although no such concerns were reported in clinical trials, long-term evidence is required to exclude adverse metabolic effects of Sac/Val.

## Data Availability

No datasets were generated or analysed during the current study.
